# Case Report: Successful management of bilateral ureteral obstruction following repair of iatrogenic urethral ligation and transection during a canine cryptorchidectomy

**DOI:** 10.3389/fvets.2026.1727528

**Published:** 2026-03-09

**Authors:** M. Haman, A. Kenzig, M. ‘t Hoen, R. Walton, J. S. Palerme, R. Stokes, K. Chappell

**Affiliations:** 1 School of Veterinary Medicine and Biomedical Sciences - Small Animal Clinical Sciences, Texas A&M University, College Station, TX, United States; 2 Department of Veterinary Clinical Sciences, Iowa State University, Ames, IA, United States; 3 Bixby Animal Specialty and Emergency Hospital, Torrance, CA, United States; 4 Memphis Veterinary Specialists & Emergency, Cordova, TN, United States; 5 Thrive Pet Healthcare Specialists North Scottsdale, Scottsdale, AZ, United States

**Keywords:** acute kidney injury, case report, cryptorchidectomy, ureteral stent, urethral transection

## Abstract

An 8-month-old male English Bulldog was presented to an academic referral center following iatrogenic urethral transection during an elective unilateral cryptorchidectomy. A contrast cystourethrogram demonstrated contrast leakage from the pre-prostatic urethra. An exploratory laparotomy confirmed ligation and transection of the pre-prostatic urethra, and a urethral anastomosis was performed. Postoperatively, the patient developed progressive azotemia, and abdominal imaging revealed bilateral hydroureter and hydronephrosis consistent with bilateral ureteral obstruction. No apparent cause of mechanical obstruction was identified during the subsequent ultrasound, cystoscopy, or laparotomy. A functional ureteral obstruction was suspected to be caused by acute, severe inflammation of the urinary bladder following the initial urethral trauma and subsequent ischemic injury, resulting in the occlusion of the ureteral papillae. Bilateral ureteral stents were surgically placed, and the azotemia resolved. Following stent placement, the dog was treated for recurrent multidrug-resistant urinary infections and urinary incontinence. The owner reported no persistent lower urinary signs during a follow-up call 46 months following the cryptorchidectomy.

## Introduction

Urethral injuries are uncommon in dogs and are reported as secondary to blunt force trauma, bite wounds, gunshots, urolithiasis, or iatrogenic causes (1–5). Treatment for urethral injuries can be conservative in cases of minor lacerations and is achieved with urinary diversion for 7–21 days via an indwelling urethral catheter or a tube cystostomy to facilitate second intention healing (6). Surgical intervention is indicated when there is disruption of 80% or more of the circumferential diameter of the urethra or if there is a significant loss of urethral tissue (5). Iatrogenic urethral transection, in which there is complete disruption of the urethral lumen, is rarely reported but most commonly occurs as a complication of cryptorchidectomy, among other surgical procedures (5, 7–11). Reported postoperative complications of urethral transection include urethral stricture, incontinence, urinary tract infection, and urethrocutaneous fistula formation (1, 2, 5, 6). The risk of stricture formation is higher in cases of complete urethral transection, and subsequently, the long-term prognosis for functional recovery remains guarded in these cases (6).

To the authors’ knowledge, bilateral ureteral obstruction has never been reported as a complication of urethral transection in dogs. The following report describes the successful treatment of functional bilateral ureteral obstruction in a dog following repair of an iatrogenic urethral ligation and transection.

## Case history

We present the case of an 8-month-old male English Bulldog who was presented to an academic referral center following an exploratory laparotomy with the intent of unilateral cryptorchidectomy. During the procedure, the pre-prostatic urethra was inadvertently ligated and transected. Upon the identification of this iatrogenic complication intraoperatively, a red rubber catheter of unknown size was placed in the urethra in retrograde fashion, confirming that the pre-prostatic urethra was completely transected and detached from the urinary bladder. The abdomen was closed routinely, and the patient was immediately referred to the academic center for further care. On presentation, the patient was mildly hypothermic with a rectal temperature of 37.1 °C (98.8 °F; reference interval (RI): 37.5–39.2 °C; 99.5–102.5 °F). All other vital signs were within normal limits. Despite the breed predilection, no clinically significant signs of brachycephalic obstructive airway syndrome (BOAS) were noted. Mild pain was elicited on abdominal palpation (Colorado State University [CSU] Pain Score: 1/4), and the urinary bladder could not readily be palpated. A 12-cm right para-preputial and ventral abdominal midline incision was present, and a single testicle was descended into the scrotum. The remainder of the physical exam was unremarkable. Admission diagnostics (Day 0), including a complete blood count (CBC), biochemistry analysis, and venous blood gas, revealed a blood urea nitrogen (BUN) level of 24 mg/dL, with a reference value of 8.57 mmol/L (RI: 10–30 mg/dL; International System of Units (SI): 2.9–10 mmol/L); a creatinine level of 1.3 mg/dL, with a reference value of 114.92 μmol/L (RI: 0.5–1.5 mg/dL; SI: 44–150 μmol/L); and a phosphorus level of 10.3 mg/dL, with a reference value of 3.33 mmol/L (RI: 2.5–6.0 mg/dL; SI: 0.9–1.8 mmol/L). A retrograde positive contrast cystourethrogram performed approximately 4 h after initial anesthetic recovery and within 2 h of referral presentation identified contrast leakage into the peritoneum between the pre-prostatic urethra and bladder neck with no contrast accumulation in the bladder, confirming urethral disruption.

**Figure 1:**
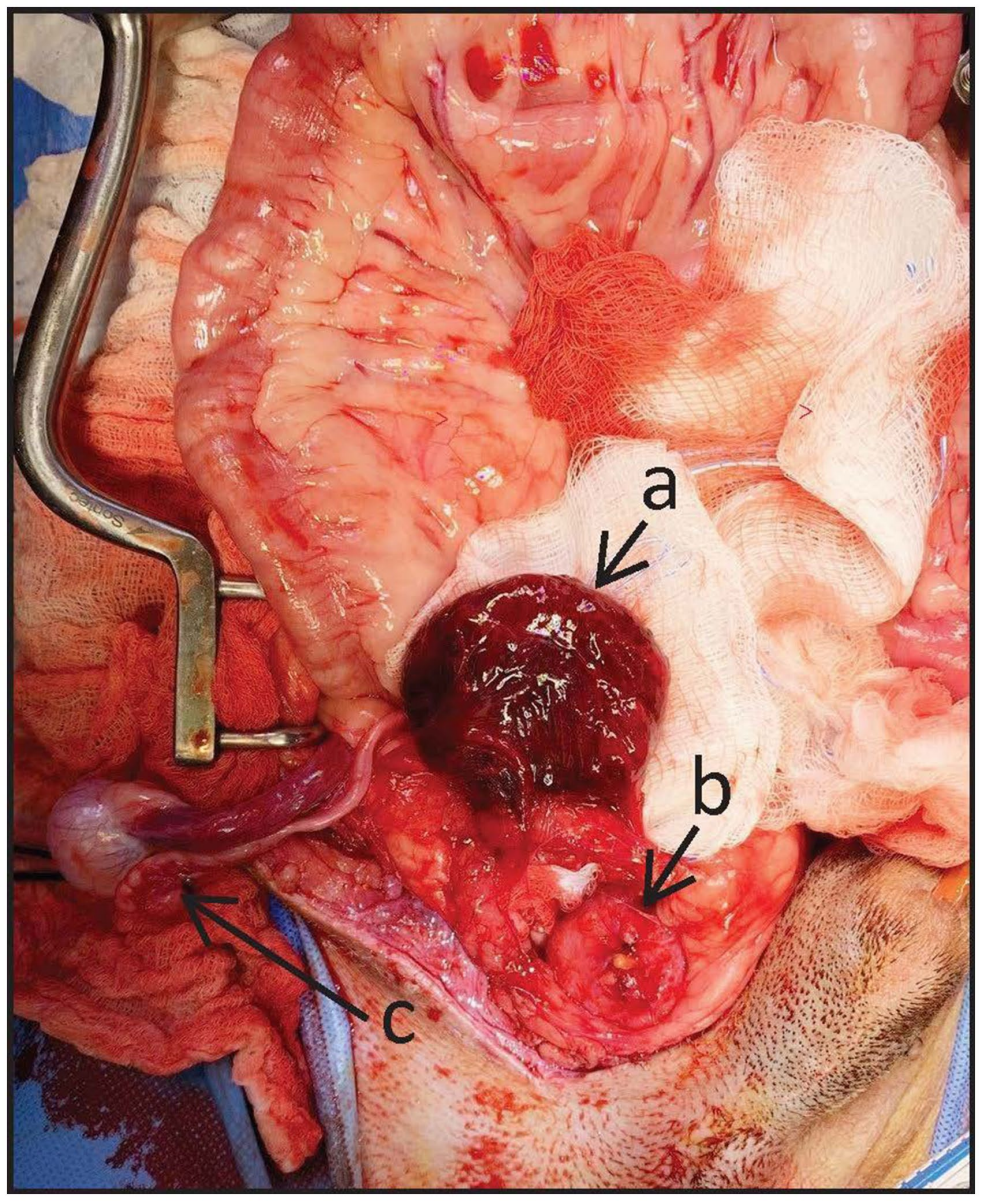
Intraoperative image, with the top of the image being cranial. **(a)** Markedly thickened and discolored urinary bladder. **(b)** Ligated and transected pre-prostatic urethra with tip of a retrograde red rubber catheter within the lumen. **(c)** Right cryptorchid testicle.

An exploratory laparotomy was subsequently performed, identifying a complete transection of the pre-prostatic urethra with one ligature proximally and one ligature distally. The left and right vas deferens were identified and noted to be intact but incorporated within the ligatures in the pre-prostatic urethra. The urinary bladder was markedly thickened with dark purple discoloration (Figure 1). Both ureters were intact and noted to be inserting normally into the trigone of the bladder, several centimeters cranial to the urethral transection site. The remainder of the visible urethra and prostate were grossly normal. The two ligatures on the urethra were removed, and a sterile 8 French red rubber catheter^1^ was passed in retrograde fashion (Figure 1) and guided across the site of transection into the urinary bladder to maintain the urethral lumen. Urethral anastomosis was performed using 5–0 Monocryl^2^ in a simple interrupted pattern. A cystotomy was not performed prior to the urethral anastomosis because the urinary bladder was severely and diffusely discolored and thickened, raising concerns that an incision of this grossly abnormal tissue could increase the risk of postoperative urinary tract incision dehiscence and subsequent uroabdomen. Following the urethral anastomosis, the red rubber catheter was removed, and an indwelling 8 French (Fr) Foley catheter^3^ was placed. The intra-abdominal right testicle was identified (Figure 1), and the spermatic cord was ligated using 2–0 PDS^4^, transected, and removed. A Jackson–Pratt drain^5^ was placed, exiting the left caudal abdominal wall, and secured with 2–0 Ethilon^6^ in a finger-trap pattern. A closed, pre-scrotal castration was performed for the left scrotal testicle using 2–0 PDS.^4^ The abdominal and pre-scrotal incisions were closed routinely, and the patient recovered uneventfully from anesthesia. Postoperatively, the patient was treated with a balanced isotonic crystalloid^7^ intravenously (IV) at a maintenance rate of 60 mL/kg/day, metoclopramide^8^ at a rate of 2 mg/kg/d IV, fentanyl^9^ at a rate of 3–5 mcg/kg/h IV, maropitant^10^ at a rate of 1 mg/kg IV (q24h), gabapentin^11^ at a rate of 11.5 mg/kg per os [PO] (q8h), and acepromazine^12^ at a rate of 0.01 mg/kg IV [pro ren nata (PRN)]. Urine output (UOP) and Jackson–Pratt drain production were monitored and recorded every 6 h.

**Figure 2:**
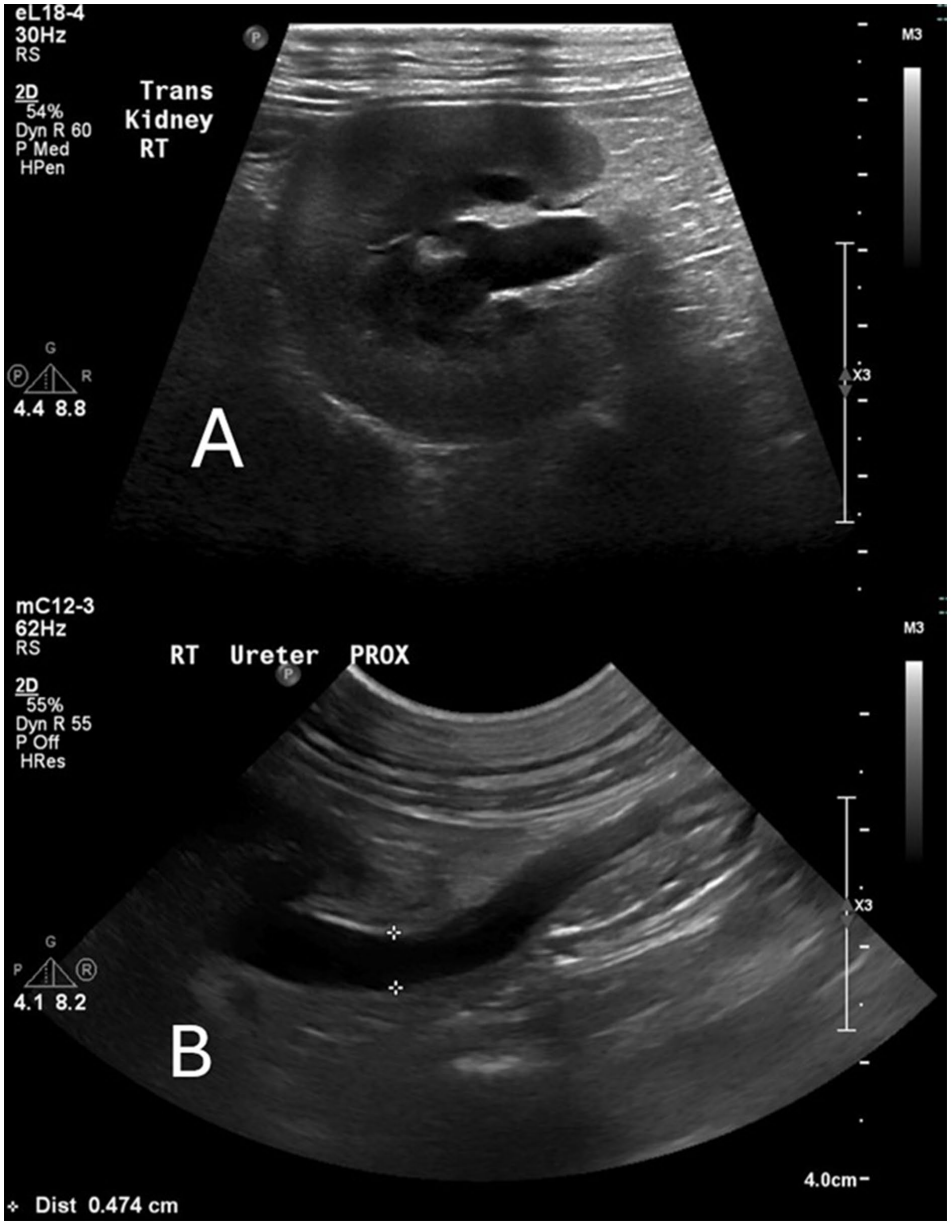
Ultrasound images from Day 3 show hydronephrosis of the right kidney and ureter. **(A)** Transverse view. **(B)** Hydroureter of the right ureter (diameter 0.474 cm).

Renal values on Day 1 postoperatively demonstrated an International Renal Interest Society (IRIS) Grade 3 acute kidney injury (AKI) [creatinine 4.2 mg/dL, 371.28 μmol/L (RI: 0.5–1.5 mg/dL; SI: 44–150 μmol/L); BUN 59 mg/dL, 21.06 mmol/L (RI: 10–30 mg/dL; SI: 2.9–10 mmol/L)] (Table 1). The rate of IV fluids with a balanced isotonic crystalloid was increased to correct an estimated 5% dehydration over 24 h to address potential pre-renal azotemia. On Day 2, the patient’s renal values progressed to an IRIS Grade 4 AKI [creatinine 6.3 mg/dL, 556.92 μmol/L (RI: 0.5–1.5 mg/dL; SI: 44–150 μmol/L); BUN 77 mg/dL, 27.49 mmol/L (RI: 10–30 mg/dL; SI: 2.9–10 mmol/L)]. The patient was clinically euhydrated on examination, so fluid therapy was reduced to a maintenance rate (approximately 60 mL/kg/d). Treatment with ampicillin–sulbactam^13^ (Unasyn, 30 mg/kg IV q8h) was initiated due to concern for potential ascending pyelonephritis, and aluminum hydroxide^14^ (35 mg/kg/d PO) was added for hyperphosphatemia [phosphorus 7.9 mg/dL, 2.6 mmol/L (RI: 2.5–6.0 mg/dL; SI: 0.8–1.9 mmol/L)]. A 10 French nasogastric (NG) tube^15^ was placed, and enteral feeding was initiated to meet resting energy requirements (RERs) with a low-fat liquid diet.^16^ On Day 3, the patient’s azotemia continued to progress [creatinine 9.0 mg/dL, 795.60 μmol/L (RI: 0.5–1.5 mg/dL; SI: 44–150 μmol/L); BUN 90 mg/dL, 32.13 mmol/L (RI: 10–30 mg/dL; SI: 2.9–10 mmol/L)]. During Days 1–3, UOP was 0.6–2.0 mL/kg/h. A urine sample was collected for culture. A cytologic examination of abdominal fluid obtained from the JP drain was consistent with sterile peritonitis. An abdominal ultrasound revealed bilateral hydroureter, hydronephrosis, and diffuse thickening of the urinary bladder wall (Figure 2). No cause for mechanical ureteral obstruction was identified. Following ultrasound, the patient was anesthetized, and cystoscopy was performed to identify the cause of bilateral hydroureters and place retrograde bilateral ureteral stents to relieve the presumed bilateral obstruction. During cystoscopy, a large blood clot was identified in the pre-prostatic urethra in addition to multiple, variably sized blood clots within the urinary bladder lumen. The ureteral papillae were not readily identified due to severe regional inflammation and blood clots. Consequently, laparotomy was pursued for ureteral stent placement since cystoscopic retrograde ureteral stent placement was not feasible. The previous abdominal incision was opened. The urinary bladder was noted to be dark red–purple in color and markedly thickened. Both ureters were identified and found to be uniformly dilated with no external cause of obstruction noted. The urethral anastomosis site was identified several centimeters caudal to the insertion of the ureters at the bladder trigone and appeared to be healing appropriately with evidence of tissue epithelialization. An approximately 3 cm ventral cystotomy was performed to allow the removal of the large blood clots noted on cystoscopy and lavage of the bladder lumen, but the ureteral papillae remained obscured and unidentifiable on gross examination due to marked mucosal discoloration and inflammation. Bilateral ureteral stents^17^ (right ureter: 18 cm 3.7 Fr; left ureter: 16 cm 4.7 Fr) were placed trans-renally in an antegrade approach using fluoroscopically guided nephrostomy and ureteropyelography. Guidewire advancement through the ureterovesical junctions was performed without difficulty or palpable impediment. Ureteropyelography failed to identify an intraluminal ureteral obstruction, suggesting obstruction at the level of the ureteral papillae. The cystotomy was closed using 2–0 PDS (see text footnote 4) in a simple continuous pattern. A new 8 Fr Foley urinary catheter was placed to leak-test the cystotomy closure; no leakage was noted. The abdomen was closed routinely. The following day (Day 4), the patient’s azotemia improved [creatinine 5.8 mg/dL, 512.72 μmol/L (RI: 0.5–1.5; SI: 44–150 μmol/L); BUN 27.13 mmol/L (RI: 10–30; SI: 2.9–10 mmol/L)]. Treatment with ampicillin–sulbactam was discontinued due to the resolution of the bilateral ureteral obstruction and negative urine culture results. The azotemia resolved on Day 7 [4 days post-stent placement; creatinine 1.2 mg/dL, 106.08 μmol/L (RI: 0.5–1.5 mg/dL; SI: 44–150 μmol/L); BUN 13 mg/dL, 4.64 mmol/L (RI: 10–30 mg/dL; SI: 2.9–10 mmol/L)].

**Table 1:** Trend in renal values during initial hospitalization (Days 0–13) following urethral anastomosis (Day 0) and placement of bilateral ureteral stents (Day 3).

Day	BUN (RI: 10–30 mg/dL)	Creatinine (RI: 0.5–1.5 mg/dL)	Phosphorus (RI: 3.2–6.0 mg/dL)	Potassium (RI: 3.9–5.3 mEq/L)
0	24	1.3	10.3	5.1
1	59	4.2	6.9	4.6
2	77	6.3	7.9	5.3
3	90	9.0	8.4	5.2
4	76	5.8	8.7	3.9
5	28	1.8	5.9	4.0
6	20	1.7	7.2	4.6
7	13	1.2	5.9	3.4
8	10	1.4		
9	14	1.3		
10	12	1.2		
11	12	1.3		
12		1.6		
13		1.4		

Following stent placement, the patient developed post-obstructive diuresis and was treated with balanced isotonic and hypotonic IV fluids^18^ to match the UOP. Intravenous potassium chloride^19^ and magnesium sulfate^20^ were supplemented as needed based on serial laboratory values. Enteral nutrition was continued via NG tube to meet total RER according to the patient’s voluntary caloric intake. On Day 9, the patient developed a fever, so the indwelling urinary catheter was removed, and a second urine culture was collected. Antibiotic therapy with enrofloxacin^21^ (10 mg/kg PO q24h) was initiated due to concern about ascending pyelonephritis. The fever resolved on Day 11. Upon the removal of the urinary catheter, the patient was noted to have urinary incontinence for which oral phenylpropanolamine^22^ (1.0 mg/kg PO q12h) was prescribed. On Day 11, antibiotic therapy was changed to doxycycline^23^ (6 mg/kg PO q12h) in accordance with urine culture susceptibility results, which demonstrated a multi-drug resistant (MDR) Enterobacter spp. urinary tract infection (UTI).

At the time of discharge on Day 13, the patient was non-azotemic (creatinine 1.4 mg/dL, 123.76 μmol/L [RI: 0.5–1.5 mg/dL; SI: 44–150 μmol/L]) but still incontinent. He was discharged with phenylpropanolamine (1.0 mg/kg PO q12h), doxycycline (6 mg/kg PO q12h), maropitant (2.4 mg/kg PO q24h PRN), capromorelin^24^ (3.0 mg/kg PO q24h PRN), and trazodone^25^ (6.0 mg/kg PO q8-12h PRN). On Day 15, the patient re-presented for the management of an AKI detected during routine follow-up with the primary veterinarian. Renal values on Day 15 demonstrated an IRIS Grade 1 AKI [creatinine 2.1 mg/dL, 185.64 μmol/L (RI: 0.5–1.5 mg/dL; SI: 44–150 μmol/L); BUN 31 mg/dL, 11.07 mmol/L (RI: 10–30 mg/dL; SI: 2.9–10 mmol/L)]. Bacteriuria and pyuria were present on urinalysis, and a third urine culture was submitted. The patient was hospitalized for 2 days and treated with IV balanced isotonic crystalloid fluids and continuation of previously prescribed medications, including doxycycline. Renal values normalized on Day 16 [creatinine 1.3 mg/dL, 114.92 μmol/L (RI: 0.5–1.5 mg/dL; SI: 44–150 μmol/L); BUN 21 mg/dL, 7.50 mmol/L (RI: 10–30 mg/dL; SI: 2.9–10 mmol/L)]. On Day 17, renal values increased [creatinine 1.7 mg/dL, 150.28 μmol/L (RI: 0.5–1.5 mg/dL; SI: 44–150 μmol/L); BUN 19 mg/dL, 6.78 mmol/L (RI: 10–30 mg/dL; SI: 2.9–10 mmol/L)] after discontinuation of IV fluids. Since the patient was eating and otherwise clinically well, he was discharged to the care of his owners on Day 17 with instructions to administer 1 L of subcutaneous balanced isotonic crystalloid fluids once daily. Urine culture results ultimately identified *Enterobacter cloacae* with resistance to doxycycline, and the patient was switched to enrofloxacin on Day 18 based on antimicrobial susceptibility. On Day 26, the patient re-presented for evaluation of a 1-day history of lethargy. Renal values were within normal limits (creatinine 1.2 mg/dL, 106.08 μmol/L [RI: 0.5–1.5 mg/dL; SI: 44–150 μmol/L]), and no cause for lethargy was identified. The patient was discharged with instructions to continue previously prescribed medications and to decrease subcutaneous fluids to 0.5 L once daily.

**Figure 3:**
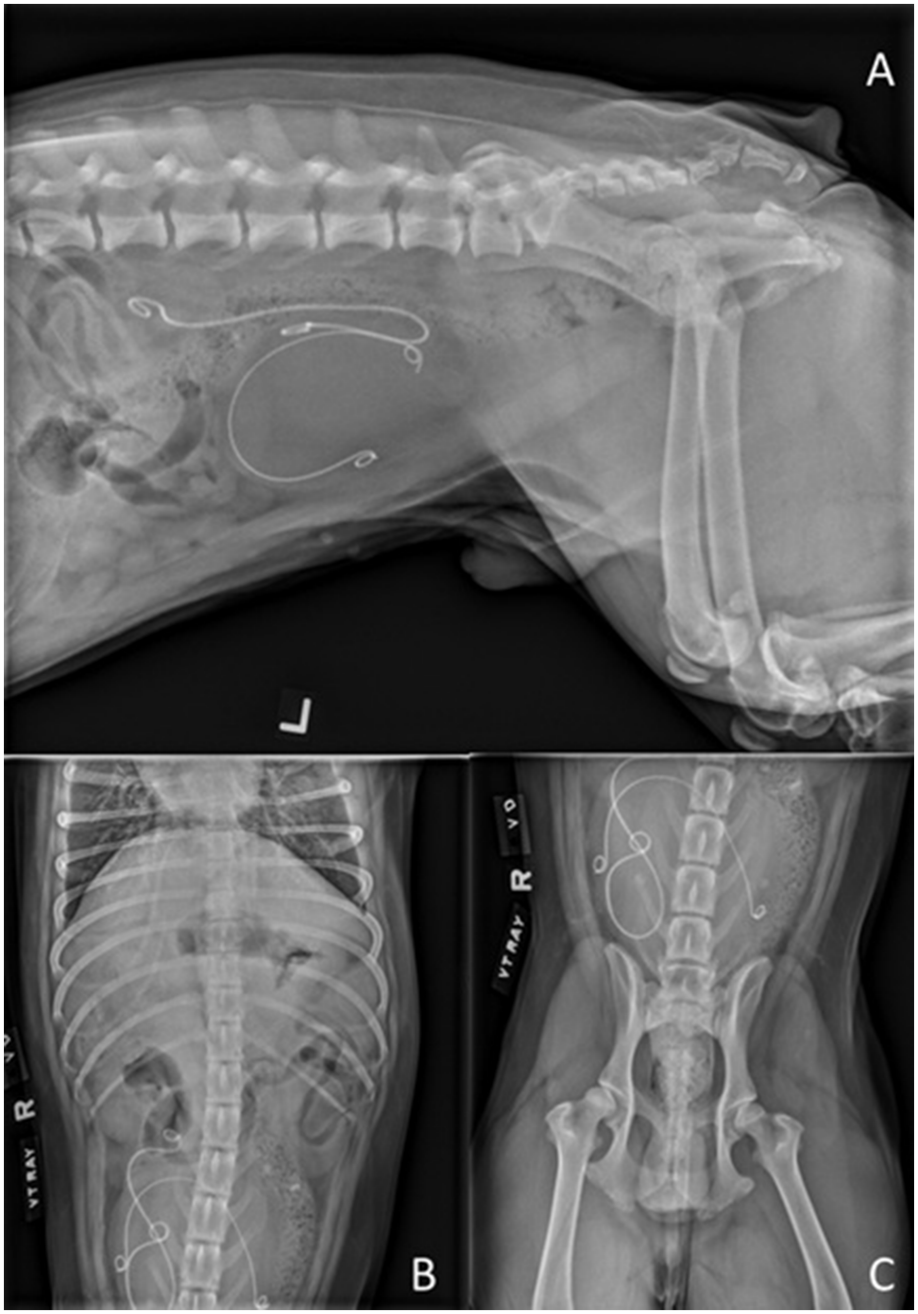
Left lateral **(A)** and VD **(B,C)** radiographs obtained on Day 63 demonstrating distal migration of both ureteral stents, with the left ureteral stent identified in the urinary bladder lumen and the right stent partially displaced to the mid-ureter.

Recurrent MDR urinary tract infections were diagnosed on Days 46 and 79 and treated with antimicrobials based on urine culture and susceptibility results. On Day 46, the owner reported that the patient’s urinary incontinence had worsened. Because the incontinence was minimally responsive to phenylpropanolamine, the drug was then discontinued. On Day 63, during a routine follow-up appointment, distal migration of both ureteral stents was identified on abdominal radiographs and ultrasound, with the left ureteral stent identified in the urinary bladder lumen and the right stent partially displaced to the mid-ureter (Figure 3). Both ureteral stents were cystoscopically removed under general anesthesia on Day 66. On Day 79, the owner reported that the patient’s urinary incontinence had improved. At the final follow-up on Day 137, the patient had stable renal values [creatinine 1.3 mg/dL, 114.92 μmol/L (RI: 0.5–1.5 mg/dL; SI: 44–150 μmol/L); BUN 21 mg/dL, 7.50 mmol/L (RI: 10–30 mg/dL; SI: 2.9–10 mmol/L)], and the owner reported that the dog was clinically normal, aside from minimal urinary incontinence at rest. At the time of article preparation, 46 months after the initial cryptorchidectomy, the owner reported via a follow-up phone call that the patient was normal at home with no urinary incontinence.

## Discussion

This is the first report of bilateral ureteral obstruction occurring in a dog following repair of an iatrogenic complication in which the urethra, rather than the ureters, was inadvertently ligated and transected. Several reports describe iatrogenic ureteral obstructions secondary to direct ligation during ovariohysterectomy or cryptorchidectomy, which did not occur in this case (7, 12–16). The bilateral ureteral obstruction in this case was identified based on concurrent severe azotemia and the ultrasonographic findings of bilateral hydronephrosis and diffuse hydroureter. The bilateral ureteral obstruction in this patient was suspected to be secondary to the initial urethral ligation and trauma, leading to subsequent ischemic injury and acute, severe, generalized inflammation of the urinary bladder, resulting in occlusion of the ureteral papillae. Ureteral obstruction has been reported due to ischemic injury in people; however, it has not been reported in veterinary medicine (17–19). Vascular supply to the distal ureters, bladder neck, and vesicoureteral junction in male dogs is provided primarily by branches of the prostatic artery, and it is possible that vascular damage occurred during the initial dissection and urethral ligation (20, 21). The urinary bladder was noted to be grossly discolored, with evidence of ischemic injury at the time of urethral anastomosis, and significant hemorrhage and tissue inflammation were identified via cystoscopy to a degree that visualization of the ureteral papillae was unachievable. These findings and the progression of severe tissue inflammation are consistent with an ischemic-reperfusion injury due to devascularization. Another potential contributing factor to the bilateral ureteral obstruction in this dog could have been mechanical obstruction by blood clots at the ureteral papillae, as the development of blood clots or hematoma in the urinary bladder is a known complication of lower urinary tract surgery (5). Multiple blood clots within the bladder lumen were confirmed to be present via cystoscopy; however, the extent to which these clots played a direct role in the ureteral obstruction cannot be fully elucidated, as visualization of the ureteral papillae was not possible during the cystoscopy. Due to the potential role the blood clots had in ureteral obstruction, a cystotomy was performed to allow for blood clot removal at the time of bilateral antegrade ureteral stent placement via laparotomy. When gross visualization of the ureteral papillae was attempted via cystotomy, they remained unidentifiable due to marked mucosal tissue discoloration and inflammation, lending credence to the theory that ischemic injury and secondary inflammation were the major components causing functional ureteral papillae obstruction. During the patient’s first laparotomy at the academic referral center, a cystotomy was not performed due to concern that incising an ischemic and inflamed urinary bladder could compromise tissue healing and increase the risk of postoperative cystotomy dehiscence and uroabdomen. During the final laparotomy, the potential risks were deemed to be outweighed by the benefits of removing blood clots, lavaging the bladder lumen, and attempting to grossly visualize the ureteral papillae. Therefore, a cystotomy was performed. These risks were mitigated by leak-testing the cystotomy prior to abdominal closure and by placing an indwelling urinary catheter to keep the bladder decompressed during initial healing.

The dog developed an IRIS Grade 4 AKI following urethral transection repair via urethral anastomosis, prompting empiric therapy and further investigation of potential pre-renal, renal, and post-renal causes. Ultimately, ultrasonographic identification of hydronephrosis and hydroureter prompted bilateral ureteral stent placement. Had an abdominal ultrasound been performed on Day 2 postoperatively, earlier detection of the developing hydronephrosis and hydroureter might have been possible; however, diagnostic imaging was not performed until continued worsening of azotemia was noted on Day 3. Bilateral ureteral stent placement on Day 3 resulted in rapid biochemical improvement. Creatinine levels decreased from 9.0 mg/dL to 1.8 mg/dL within 2 days and normalized to 1.2 mg/dL within 4 days of stent placement. This response is consistent with relief of post-renal azotemia and strongly supports functional obstruction as the primary cause of this patient’s AKI. Both stents eventually migrated and were ultimately removed. However, the patient remained non-azotemic with no apparent recurrence of ureteral obstruction. Therefore, transitory bilateral ureteral obstruction due to severe ischemic injury, causing generalized bladder inflammation and subsequent functional obstruction of the ureteral papillae, as well as a possible additional component of mechanical obstruction secondary to intraluminal blood clots, likely caused the AKI in this patient. In future cases of iatrogenic trauma and suspected ischemic injury to the lower urinary tract, where marked discoloration and bladder tissue thickening are noted without specific trauma or ureteral abnormalities, pre-emptive ureteral stent placement could be considered to maintain ureteral papillae patency during subsequent reperfusion and inflammation.

## Conclusion

This case report describes a novel complication of iatrogenic urethral trauma and the use of temporary ureteral stenting to treat acute ureteral obstruction following repair of iatrogenic trauma to the pre-prostatic urethra. Placement of bilateral ureteral stents during surgical correction of urethral trauma should be considered on a case-by-case basis.

## Data Availability

The original contributions presented in the study are included in the article/supplementary material, further inquiries can be directed to the corresponding author.

## Footnotes

1 Red rubber catheter, Covidien LLC, Mansfield, MA.

2 Monocryl, Ethicon, Johnson and Johnson, San Lorenzo.

3 Foley urinary catheter, Cook Medical, Bloomington, IN.

4 2-0 PDS, Ethicon, Johnson and Johnson, San Lorenzo.

5 Jackson Pratt drain, Covidien LLC, Mansfield, MA.

6 2-0 Ethilon, Ethicon, Johnson and Johnson, San Lorenzo.

7 Lactated ringers, B. Braun, Irvine, CA.

8 Fentanyl citrate, Hospira Inc., Lake Forest, IL.

9 Metoclopramide HCl, Pfizer Inc., New York, NY.

10 Maropitant, Zoetis LLC, Kalamazoo, MI.

11 Gabapentin, Actavis Elizabeth LLC, Elizabeth, NJ.

12 Acepromazine maleate, Boehringer Ingelheim Vetmedica, Inc., St. Joseph, MO.

13 Ampicillin-sulbactam, Auromedics Pharma LLC, E. Windsor, NJ.

14 Aluminum hydroxide (Phos-Bind) powder, Rx Vitamins, Oldsmar, FL.

15 Nasogastric tube, MILA International, Inc., Hebron, KY.

16 Royal Canin GI low-fat liquid diet, Royal Canin, St. Charles, MO.

17 Ureteral stents, Infiniti Medical, Palo Alto, CA.

18 0.45% NaCl solution, B. Braun, Irvine, CA.

19 Potassium chloride injectable, Hospira Inc., Lake Forest, IL.

20 Magnesium sulfate, Fresenius Kabi, Lake Zurich, IL.

21 Enrofloxacin (Baytril injectable), Bayer Healthcare LLC, Shawnee Mission, KS.

22 Phenylpropanolamine tablets, Pegasus Laboratories, Inc., Pensacola, FL.

23 Doxycycline hyclate, Teva Pharmaceuticals USA, Inc., Parsippany, NJ.

24 Capromorelin (Entyce) oral solution, Elanco Animal Health, Greenfield, IN.

25 Trazodone, Major Pharmaceuticals, Livonia, MI.
